# Aerosol assisted fabrication of two dimensional ZnO island arrays and honeycomb patterns with identical lattice structures

**DOI:** 10.3762/bjnano.1.9

**Published:** 2010-11-22

**Authors:** Mitsuhiro Numata, Yoshihiro Koide

**Affiliations:** 1 Department of Material and Life Chemistry, Faculty of Engineering, Kanagawa University, 3-27-1 Rokkakubashi, Yokohama, Kanagawa-ku, Kanagawa 221-8686 Japan; Fax +81-45-413-9770, Tel +81-45-481-5661

**Keywords:** aerosol, photonic crystal, polystyrene bead, TiO_2_, ZnO

## Abstract

Two dimensional island arrays and honeycomb patterns consisting of ZnO nanocrystal clusters were fabricated on predefined TiO_2_ seed patterns prepared by vacuum free, aerosol assisted wet-chemical synthesis. The TiO_2_ seed patterns were prepared by applying an aerosol of a water soluble titanium complex on hexagonally close-packed polystyrene bead arrays for different lengths of time. Scanning electron microscopy revealed that a dot array grows into a honeycomb shape as increasing amounts of the precursor were deposited. ZnO nucleation on substrates with a dot array and honeycomb patterns resulted in the formation of two discrete patterns with contrasting fill fractions of the materials.

## Findings

The wide bandgap (3.37 eV) and large exciton-binding energy (60 meV) of ZnO renders it a promising candidate for inexpensive transparent conductors and for optical applications at room temperature. A large-scale, high throughput, size-controlled assembly of ZnO nanostructures would allow the fabrication of a number of conceivable optoelectronic devices, such as dye sensitized solar cells [1–2], electronic sensors [3], UV lasers [4–5], photocatalysts [6], and two-dimensional photonic crystals (PhCs) [7–9]. To this end, a number of techniques, including an atomic layer epitaxy [10], chemical vapor deposition [11], and photoresist patterning [12–13], have been used to deposit various morphologies of ZnO patterns on substrates.

There is intense current interest in the manipulation of light using photonic band gaps (PBG) created in an air-hole type 2D PhC, in which a material with relatively high refractive indices are periodically arranged on a flat substrate in open space. For example, PBGs have been observed with arrays of spherical polystyrene, silica, protein, etc. [14–16]. Polymer sphere lithography (PSL) is a soft lithographic method that enables deposition of vaporized materials in periodic arrangement using triangular apertures formed by hexagonally close-packed polystyrene colloidal crystals as masks [17–19]. For example, Li et al. prepared patterned arrays of ZnO nanopillars by defining the growth sites and spaces using PSL [20–21]. However, because (i) the resulting nanostructures have triangular shapes with vertices alternatively pointing in opposite directions and (ii) the spatial pitch is uneven, a conventional PSL procedure is incompatible with 2D PhC structure, which requires the presence of a regular array of a uniform structure [22–23].

In the present study, we combined PSL with mist chemical vapor deposition (m-CVD) to demonstrate the fabrication of two discrete patterns, a dot array and a honeycomb structure, having the identical spatial pitch but different fill fractions of periodic ZnO nanocrystal clusters on a 1.5 cm × 1.5 cm n-type (100) silicon wafer. In a newly developed m-CVD technique, an aqueous solution of a water soluble titanium complex (TiO_2_ precursor) [24–25] was delivered as aerosol on a polystyrene bead monolayer on SiO_2_/Si substrates. Subsequent pyrolysis converts the precursor to crystalline TiO_2_ with concomitant removal of the polystyrene. The resulting TiO_2_ seed pattern was then used to direct ZnO nanocrystals growth in a one-pot solution process [2,9,26–27]. In addition, this type of laminated structure is considered advantageous in achieving the large aspect ratio necessary to observe the photonic bandgap via surface reflection measurements [9].

**Figure 1:**
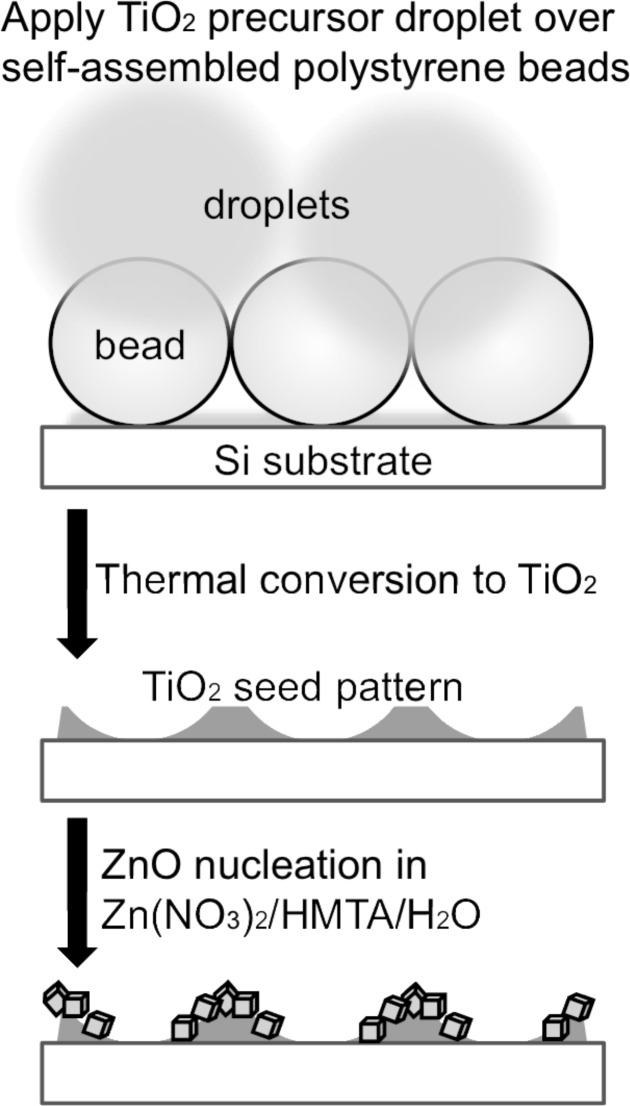
Step-by-step representation of a PSL/m-CVD approach to the patterned nucleation of ZnO nanocrystal clusters.

Figure 1 summarizes the ZnO nanocrystal array fabrication under the PSL/m-CVD procedures. First, a polystyrene beads (median diameter = 1.50 μm) monolayer was prepared as previously described [22]. A fine spray mist of TiO_2_ precursor (50 mM) was delivered to the substrate surface by an ultrasonic nebulizer (air flow rate = 7 L/min, median droplet size = 1.9 μm) in a downward direction on the monolayer placed in a vessel (30 (height) × 15 (width) × 15 (depth) cm^3^). After drying for 1 h in air, calcination was performed at atmospheric pressure in an oven set at 500 °C for 2 h. Subsequently, ZnO nanocrystals were grown by immersing the substrate in a cylindrical glass vessel that contained a mixed aqueous solution of zinc nitrate hexahydrate (10 mM) and hexamethylenetetramine (10 mM). The substrate was held in the solution with the TiO_2_ patterned face downwards and heated on a hot plate at 90 °C for 90 min unless otherwise mentioned.

**Figure 2:**
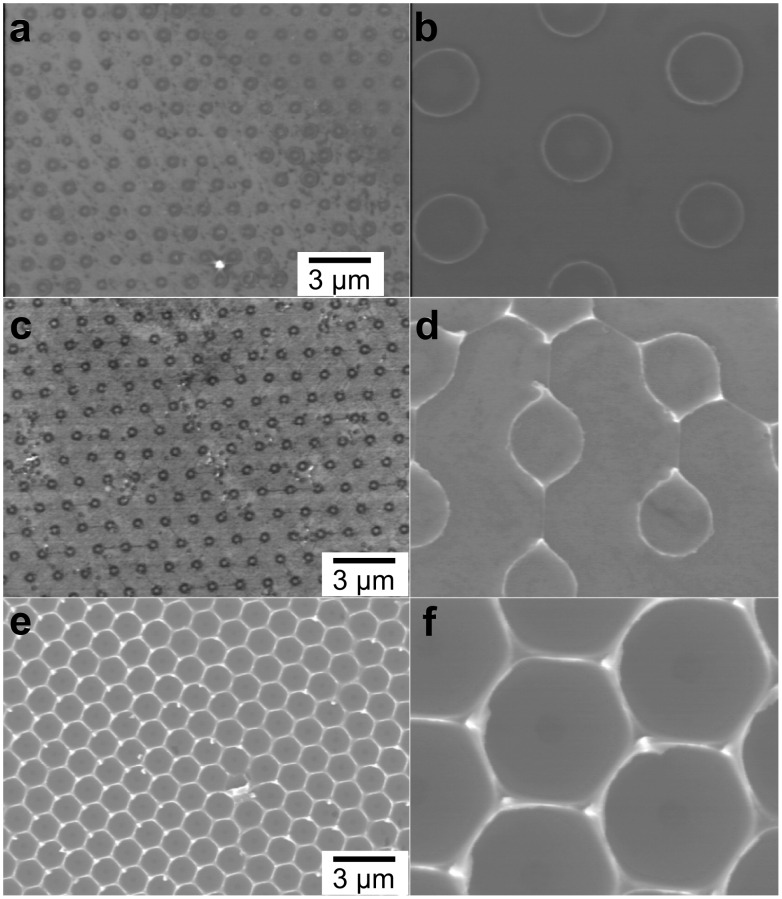
Low- (left column) and high-magnification (right column) SEM images of various TiO_2_ seed patterns created after deposition of TiO_2_ precursor for 10 s (a, b), 20 s (c, d), and 60 s (e, f).

The progressive development of a TiO_2_ seed pattern was observed by scanning electron microscopy (SEM) (Figure 2). A sample prepared by applying the aerosol for 10 s formed an array of TiO_2_ convex dots on the surface (Figure 2a and Figure 2b). The maximum diameter measured at the vertex (light colored ring) was approximately 0.63 μm which corresponds to the estimated height of 70 nm. A longer spray time (20 s) resulted in the formation of larger, interconnected convex dots with a diameter of 0.73 μm (estimated height of 95 nm) (Figure 2c and Figure 2d). A further increase in the spray time (60 s) resulted in the formation of a honeycomb pattern (Figure 2e and Figure 2f), in which the maximum diameter of each hemisphere was about 1.36 μm (estimated height of 430 nm). These observations suggest that the TiO_2_ precursor droplet, which has a larger diameter than a polystyrene bead, wet the upper hemisphere and stream down the surfaces and then build up at around the pivot. As the volume of the precursor increases, the convex dots grow larger and finally come together to form a honeycomb structure.

**Figure 3:**
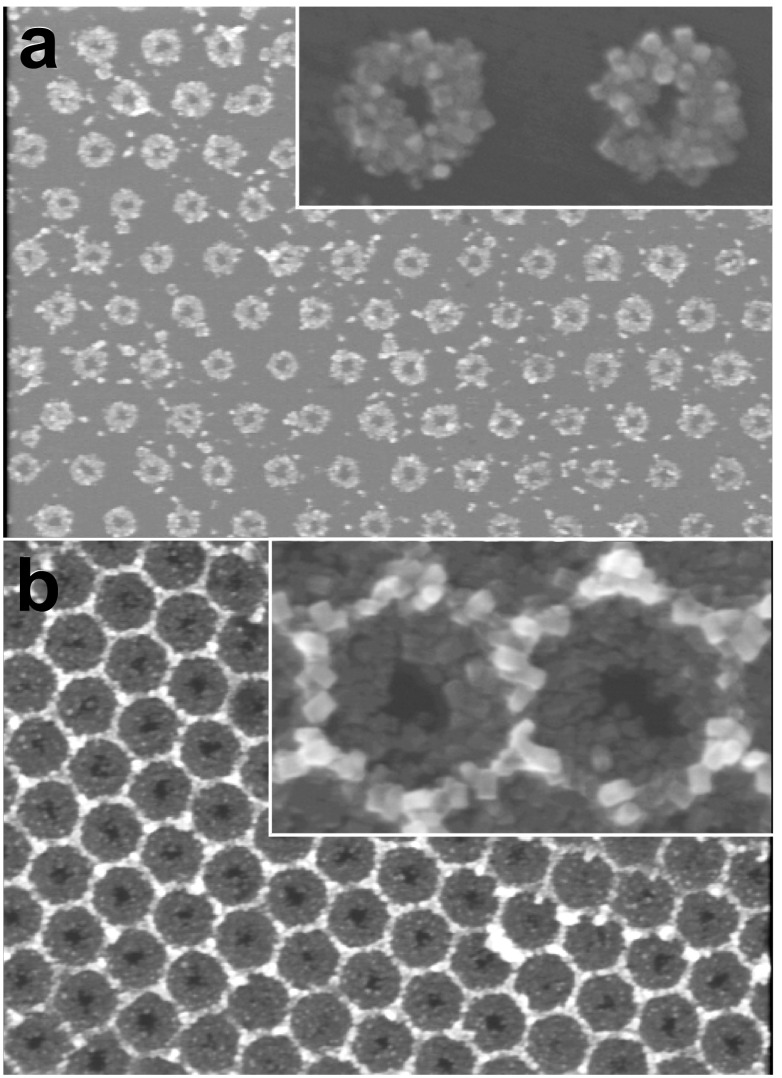
SEM images of ZnO nanocrystal clusters nucleated in shapes of an island array (a) and a honeycomb (b) on TiO_2_ seed patterns comparable to those in Figure 2a and Figure 2e, respectively. The insets show the magnified portions of each image.

**Figure 4:**
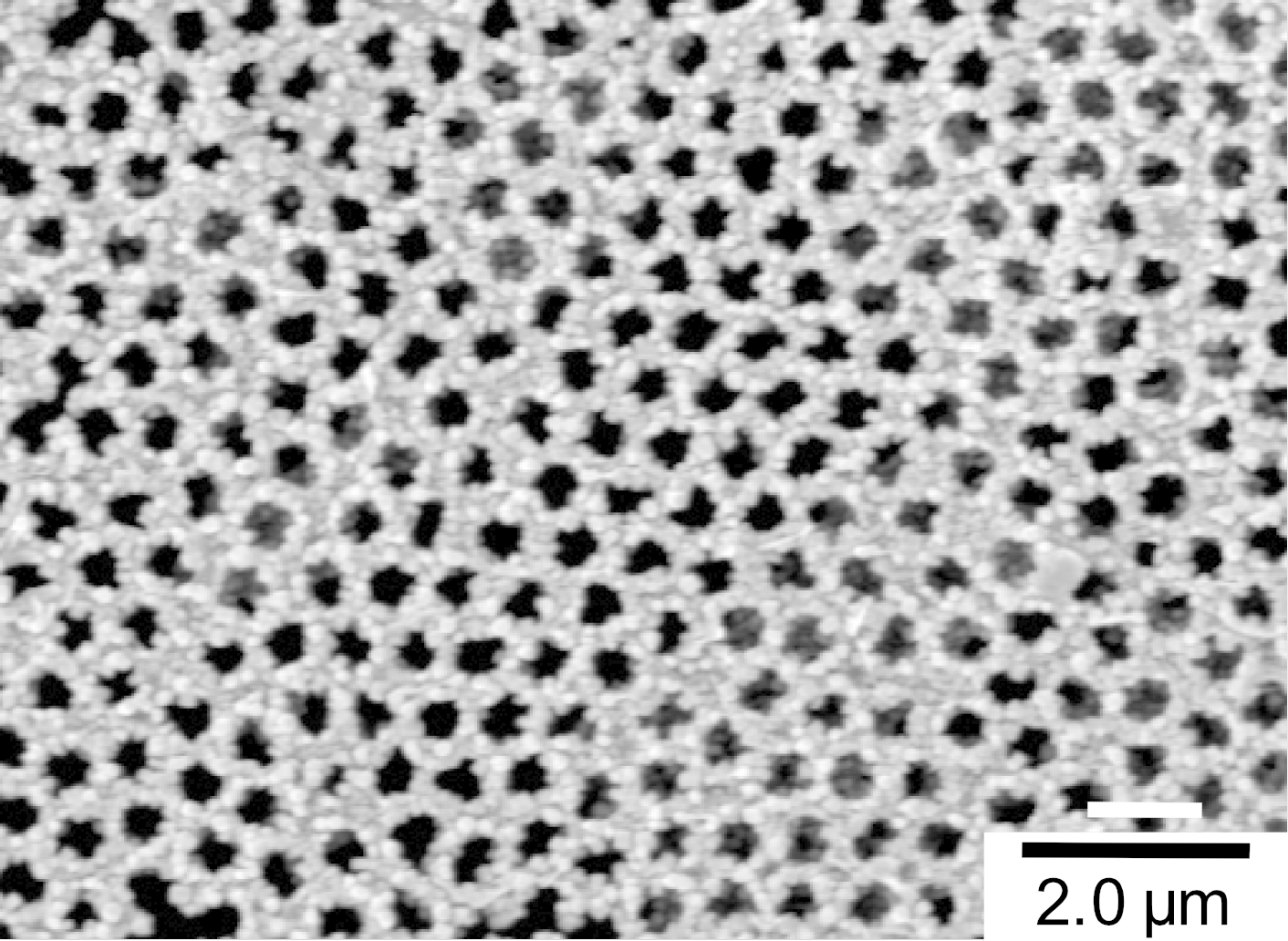
SEM image of ZnO nanocrystal clusters nucleated on a honeycomb pattern with the spatial pitch of 500 nm.

Figure 3 shows ZnO nanocrystal clusters nucleated in shapes of an island array (Figure 3a) and a honeycomb structure (Figure 3b) using the seed patterns comparable to those shown in Figure 2a and Figure 2e, respectively. In both cases, square-disc and cubic ZnO crystals with a grain size finer than 100 nm adhered almost exclusively on the seed regions and thus lead to different fill fractions of the materials (i.e., ZnO and air). The Scotch tape test was conducted to gauge the adhesive strength of the ZnO nanocrystals adhered to the TiO_2_ seeds which revealed that the crystals were hardly removed by the tape applied to the surface with light pressure. The control of the spatial pitch was demonstrated as well by changing the diameter of the polystyrene beads employed in the PSL procedure. Figure 4 shows the ZnO honeycomb structure grown on a TiO_2_ seed pattern prepared on 500 nm beads. This result proves that PSL/m-CVD can direct liquid precursors and control the size and pitch of patterns, be it a dot array or a honeycomb shape, by simply choosing the right combination of the applied volume and a bead size.

In summary, two contrasting patterns of ZnO nanocrystal clusters were readily fabricated on SiO_2_/Si substrates by using a PSL/m-CVD method. This new method has two major advantages: firstly it allows fabrication of regularly spaced, size-controlled dot arrays of TiO_2_ seed patterns that ultimately are able to be transformed to honeycomb structures and secondly fabrication of variable fill fractions of materials will be a valuable technique for systematic manipulation of the PBG [28].

## References

[1] Yuhas B D, Yang P (2009). J Am Chem Soc.

[2] Wang M, Huang C, Cao Y, Yu Q, Guo W, Huang Q, Liu Y, Huang Z, Huang J, Wang H (2009). Appl Phys Lett.

[3] Fan H J, Lee W, Hauschild R, Alexe M, Le Rhun G, Scholz R, Dadgar A, Nielsch K, Kalt H, Krost A (2006). Small.

[4] Chang Y-C, Yang W-C, Chang C-M, Hsu P-C, Chen L-J (2009). Cryst Growth Des.

[5] Zhang J-Y, Zhang Q-F, Deng T-S, Wu J-L (2009). Appl Phys Lett.

[6] Lin C-J, Lu Y-T, Hsieh C-H, Chien S-H (2009). Appl Phys Lett.

[7] Fu M, Zhou J, Yu J (2010). J Phys Chem C.

[8] Matsuu M, Shimada S, Masuya K, Hirano S, Kuwabara M (2006). Adv Mater.

[9] Wang X, Neff C, Graugnard E, Ding Y, King J S, Pranger L A, Tannenbaum R, Wang Z L, Summers C J (2005). Adv Mater.

[10] Yan M, Koide Y, Babcock J R, Markworth P R, Belot J A, Marks T J, Chang R P H (2001). Appl Phys Lett.

[11] Wang A, Dai J, Cheng J, Chudzik M P, Marks T J, Chang R P H, Kannewurf C R (1998). Appl Phys Lett.

[12] Greyson E C, Babayan Y, Odom T W (2004). Adv Mater.

[13] Lai Y, Lin Z, Huang J, Sun L, Chen Z, Lin C (2010). New J Chem.

[14] Matsushita S I, Shimomura M (2004). Chem Commun.

[15] Wang W, Gu B, Liang L, Hamilton W (2003). J Phys Chem B.

[16] Reese C E, Guerrero C D, Weissman J M, Lee K, Asher S A (2000). J Colloid Interface Sci.

[17] Ryu K, Badmaev A, Gomez L, Ishikawa F, Lei B, Zhou C (2007). J Am Chem Soc.

[18] Hulteen J C, Van Duyne R P (1995). J Vac Sci Technol, A.

[19] Haynes C L, Van Duyne R P (2001). J Phys Chem B.

[20] Li C, Hong G, Wang P, Yu D, Qi L (2009). Chem Mater.

[21] Liu D F, Xiang Y J, Wu X C, Zhang Z X, Liu L F, Song L, Zhao X W, Luo S D, Ma W J, Shen J (2006). Nano Lett.

[22] Koide Y, Fujisawa K, Nakane M (2008). Colloids Surf, A.

[23] Nam H J, Jung D-Y, Yi G-R, Choi H (2006). Langmuir.

[24] Kobayashi M, Petrykin V V, Kakihana M, Tomita K, Yoshimura M (2007). Chem Mater.

[25] Tomita K, Petrykin V, Kobayashi M, Shiro M, Yoshimura M, Kakihana M (2006). Angew Chem, Int Ed.

[26] Wang N, Sun C, Zhao Y, Zhou S, Chen P, Jiang L (2008). J Mater Chem.

[27] Lee C-Y, Wang J-Y, Chou Y, Liu M-Y, Su W-F, Chen Y-F, Lin C-F (2010). J Appl Phys.

[28] Song B-S, Noda S, Asano T (2003). Science.

